# Long‐term outcomes of standardized colonic stenting using WallFlex as a bridge to surgery: Multicenter prospective cohort study

**DOI:** 10.1111/den.14137

**Published:** 2021-10-01

**Authors:** Toshio Kuwai, Yuzuru Tamaru, Ryusaku Kusunoki, Shuntaro Yoshida, Takeaki Matsuzawa, Hiroyuki Isayama, Iruru Maetani, Mamoru Shimada, Tomonori Yamada, Shuji Saito, Masafumi Tomita, Koichi Koizumi, Toshiyasu Shiratori, Toshiyuki Enomoto, Yoshihisa Saida

**Affiliations:** ^1^ Department of Gastroenterology National Hospital Organization Kure Medical Center and Chugoku Cancer Center Hiroshima Japan; ^2^ Department of Endoscopy and Endoscopic Surgery Graduate School of Medicine The University of Tokyo Tokyo Japan; ^3^ Department of Gastroenterology Graduate School of Medicine Juntendo University Tokyo Japan; ^4^ Division of Gastroenterology and Hepatology Department of Internal Medicine Tokyo Japan; ^5^ Department of Surgery Toho University Ohashi Medical Center Tokyo Japan; ^6^ Department of Gastroenterology Tokyo Metropolitan Cancer and Infectious Disease Center Komagome Hospital Tokyo Japan; ^7^ Department of Surgery Imusumiyoshi General Hospital Saitama Japan; ^8^ Department of Surgery Toyonaka Keijinkai Hospital Osaka Japan; ^9^ Department of Gastroenterology Japanese Red Cross Nagoya Daini Hospital Aichi Japan; ^10^ Division of Surgery Gastrointestinal Center Yokohama Shin‐Midori General Hospital Kanagawa Japan; ^11^ Department of Surgery Kobe Tokushukai Hospital Hyogo Japan; ^12^ Department of Gastroenterology Kameda Medical Center Chiba Japan

**Keywords:** bridge to surgery, colorectal cancer, perforation, self‐expandable metallic stent, stenting

## Abstract

**Objectives:**

The oncological outcomes, especially high recurrence rate, of bridge‐to‐surgery (BTS) self‐expandable metallic stent (SEMS) placement remain concerning, emphasizing the necessity of standardized SEMS placement. However, its impact on long‐term BTS outcomes is unknown. We investigated the long‐term outcomes of BTS colonic stenting using standardized SEMS placement.

**Methods:**

This prospective, multicenter cohort study conducted at 46 hospitals in Japan (March 2012 to October 2013) included consecutive patients with stage II and III obstructive colorectal cancer managed with BTS SEMS placement. The SEMS placement technique was standardized by information dissemination among the participating hospitals. The primary outcome was overall survival (OS) after SEMS placement, and the secondary outcomes were relapse‐free survival (RFS), recurrence, and short‐term outcomes of SEMS placement and surgery.

**Results:**

The 1‐, 3‐, and 5‐year OS rates were 94.1%, 77.4%, and 67.4% (Kaplan–Meier), respectively, with high technical success (99.0%, 206/208) and low perforation (1.9%, 4/208) rates. The 1‐, 3‐, and 5‐year RFS rates were 81.6%, 65.6%, and 57.9% (Kaplan–Meier), respectively, and the overall recurrence rate was 31.0% (62/200). The RFS rate was significantly poorer in patients with perforation (*n* = 4) than in those without perforation (*n* = 196) (log‐rank *P =* 0.017); moreover, perforation was identified as an independent factor affecting RFS (hazard ratio 3.31; 95% confidence interval 1.03–10.71, multivariate Cox regression).

**Conclusion:**

This large, prospective, multicenter study revealed satisfactory long‐term outcomes of BTS colonic stenting using a standardized SEMS insertion method, which might be specifically due to the reduced perforation rate. (UMIN000007953).

## INTRODUCTION

Nearly 10% of patients with colorectal cancer present with large bowel obstruction.^1^, ^2^ In Japan, colonic self‐expandable metallic stent (SEMS) placement has been used not only for palliation but also as a bridge‐to‐surgery (BTS) approach in the management of obstructive colorectal cancer.^2^, ^3^ BTS colonic stenting is more advantageous than emergency surgery (ES) owing to improved short‐term outcomes, including primary anastomosis, stoma creation, and complications.^4^, ^5^, ^6^

Despite these advantages, concerns regarding the long‐term outcomes of BTS colonic stenting have been raised,^7^ likely owing to the lack of consensus on oncological outcomes after SEMS placement. Adverse events caused by SEMS placement, especially perforation, have been considered major contributors to poor oncological outcomes.^8^ Although the European Society of Gastrointestinal Endoscopy updated their guidelines to basically recommend BTS colonic stenting in 2020, the same body noted concerns regarding high recurrence rates after BTS colonic stenting, emphasizing the necessity for standardized SEMS placement to prevent adverse events.^9^ However, currently no study has evaluated the long‐term outcomes of BTS colonic stenting with a standardized method, characterized by high technical success rate with low perforation rate, in large, multicenter, prospective cohort study settings.

In 2012, the Japan Colonic Stent Safe Procedure Research Group (JCSSPRG), organized within the Japan Gastroenterological Endoscopy Society to provide instructions on safety procedures for colonic SEMS placement, provided recommendations on adequate SEMS placement methods. Standards were shared among participating hospitals before the start of this study. This led to a multicenter, prospective cohort study that aimed to determine the long‐term outcomes of BTS colonic stenting for stage II and III obstructive colorectal cancers in a large cohort using a standardized SEMS placement method.

## METHODS

### Study design and participants

This prospective, multicenter clinical study was conducted by JCSSPRG to assess the efficacy and safety of colonic stenting from March 2012 to October 2013 at 46 sites across Japan, including 14 academic and 32 community hospitals, in accordance with the principles of the Declaration of Helsinki. The study was registered with the University Hospital Medical Information Network Clinical Trial Registry (UMIN000007953). Institutional Review Boards of the participating hospitals granted approval prior to study initiation, and informed consent for SEMS placement and registration of clinical data for research use was obtained from the patients. The participating institutions registered all patients with acute colorectal obstruction managed using WallFlex enteral colonic stents (Boston Scientific, Marlborough, MA, USA) during the entire study period, and all clinical data were prospectively collected.

Before the study, JCSSPRG conducted a workshop to discuss SEMS placement, in which several experienced physicians presented their experiences in developing safe SEMS placement procedures. A workshop summary was subsequently uploaded to the study website,^10^ containing standard SEMS placement methods based on previously published data,^11^, ^12^ and guidance on procedure‐related technical details to achieve procedure standardization among the participating hospitals. The previously reported short‐term outcomes of the original study showed excellent success rates,^13^, ^14^, ^15^ and here we report the long‐term outcomes.

### Inclusion and exclusion criteria

Inclusion criterion for enrollment in the original study was the presence of malignant colonic obstruction diagnosed using abdominal radiography, colonoscopy, or computed tomography. We excluded patients with a benign stricture, enteral ischemia, suspected or impending perforation, intra‐abdominal abscess, contraindication to endoscopic treatment, previous colonic SEMS placement, and any use of the stent which was not indicated.

**Figure 1:**
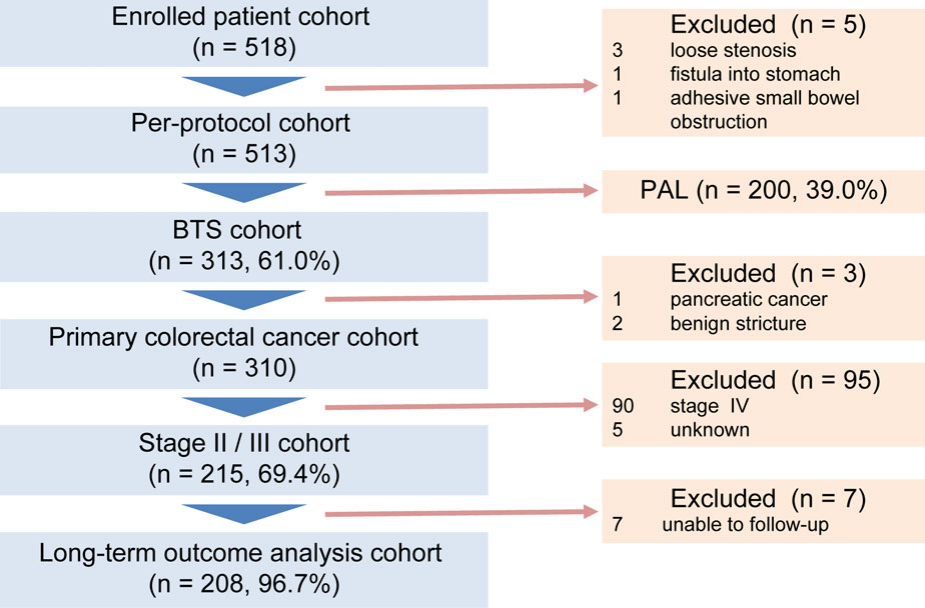
Schematic of the study flow. BTS, bridge‐to‐surgery; PAL, palliation.

From the available cohort, we only included patients diagnosed with primary stage II or III colorectal cancer and those who had undergone BTS SEMS placement (Fig. 1).

### SEMS placement procedures

An uncovered WallFlex enteral colonic stent was used in all patients. The detailed colonic SEMS placement procedure was presented on the study website^10^ and shared among participating facilities before the study (Appendix S1).

The following were the major points of the procedure. The procedure was performed in a fluoroscopy room, and positioning using the colonoscope during SEMS placement was accomplished under radiographic guidance; furthermore, prior to SEMS placement, a guidewire was advanced through a sheath using the endoscopic retrograde cholangiopancreatography technique. Balloon or bougie dilatation of the stenotic region was not performed, the distal end of the stenotic lesion was marked with metal clips, and prophylactic SEMS placement was not performed.

### BTS procedures

If no adverse events were observed after SEMS placement, the patients were discharged or made to rest in the hospital for clinical improvement. The surgical timing was based on clinical status, adverse events, and resolution of colon distension. Experienced colorectal surgeons with preference for less invasive laparoscopic techniques performed all surgical resections. The need for adjuvant chemotherapy was determined at institutional, multidisciplinary oncology meetings. At all timepoints, patients were treated with standard treatment regimens and followed for 5 years after SEMS placement based on the best available data and the Japanese Society for Cancer of the Colon and Rectum guidelines.^3^, ^16^

### Outcomes and definitions

The primary outcome was overall survival (OS), defined as the time from SEMS placement to death from any cause or to the last date of contact with the surviving patient. The secondary outcomes were relapse‐free survival (RFS), recurrence rates, and short‐term outcomes of SEMS placement and surgery.

Self‐expandable metallic stent placement prior to scheduled elective resection of primary tumors, regardless of the duration between SEMS insertion and surgery, was classified as a BTS procedure. SEMS placement was classified as a palliative procedure in patients without scheduled surgery; these patients were excluded from this study.

Technical success was defined as stent deployment across the entire stricture length on the first attempt without adverse events. Clinical success was defined as sustained relief of obstructive symptoms from the time of stent placement to surgery without any stent‐related adverse events or the need for endoscopic reintervention or ES. The ColoRectal Obstruction Scoring System (CROSS) was used to assess the oral intake level and abdominal symptoms before the procedure.^13^

### Statistical analysis

Data on baseline characteristics and clinical, tumor, and surgery‐related characteristics were expressed as mean ± standard deviation, median (range, interquartile range [IQR]), or percentages. Participants with missing data and those who failed to complete follow‐up were excluded from the analysis. Survival rates were calculated using the Kaplan–Meier method and compared using the log‐rank test. Univariate and multivariate Cox regression analyses were performed to identify the factors affecting RFS. The following variables were included: age, sex, performance status (PS), CROSS, tumor location, tumor–node–metastasis (TNM) staging, surgical approach, postoperative complication, and perforation. The explanatory variables were selected using a stepwise procedure (include *P* < 0.10 and exclude *P* > 0.20). Statistical analyses were performed using JMP v. 15 (SAS Institute, Cary, NC, USA).

## RESULTS

### Baseline characteristics of the study cohort

Among 518 consecutive patients enrolled in the original study, BTS stenting was performed in 313 patients, including stage II or III colorectal cancer in 215. After excluding seven patients lost to follow‐up, we analyzed 208 patients (Fig. 1).

All patients were Japanese, with a median age of 72 (range 25–94; IQR 62–79) years (Table 1). CROSS scores were 0, 1, 2, 3, and 4 in 77, 68, 20, 30, and 13 patients, respectively, and 96 (46.2%) and 112 (53.8%) patients had stage II and III colorectal cancer, respectively.

**Table 1:** Baseline patient demographics and tumor characteristics (*n* = 208)

Age, years, median (range, IQR)	72 (25–94, 62–79)
Sex, *n* (%)	
Male	112 (53.8)
Female	96 (46.2)
PS, *n* (%)	
0 or 1	180 (86.5)
2–4	28 (13.5)
CROSS, *n* (%)	
0	77 (37.0)
1	68 (32.7)
2	20 (9.6)
3	30 (14.4)
4	13 (6.3)
Tumor location, *n* (%)	
Rectum	7 (3.4)
Left side of the colon	148 (71.1)
Right side of the colon	53 (25.5)
TNM staging, *n* (%)	
II	96 (46.2)
IIA	72 (34.6)
IIB	19 (9.1)
IIC	5 (2.4)
III	112 (53.8)
IIIA	0 (0)
IIIB	74 (35.6)
IIIC	38 (18.3)

CROSS, ColoRectal Obstruction Scoring System; IQR, interquartile range; PS, performance status; TNM, tumor–node–metastasis.

### Short‐term outcomes

**Figure 2:**
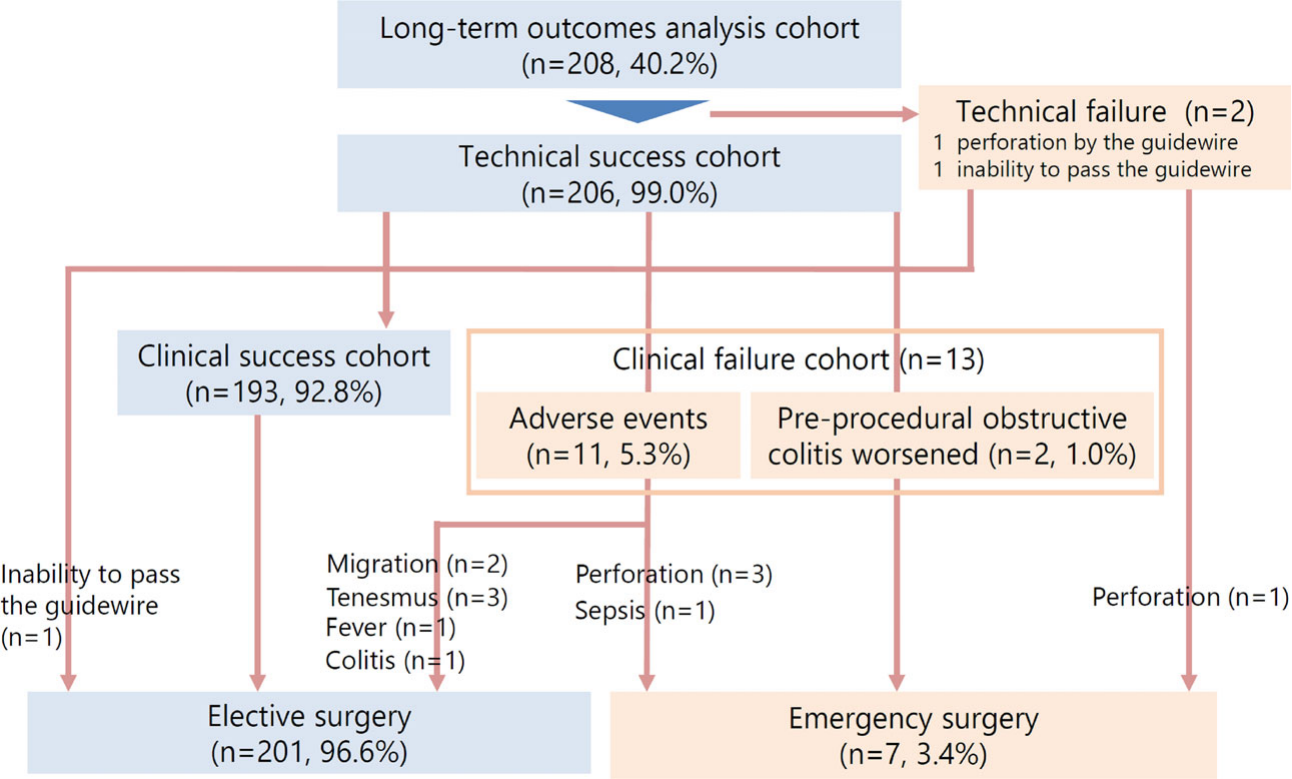
Schematic of the flow of patients receiving colonic stents as a bridge‐to‐surgery.

The technical and clinical success rates with BTS SEMS placement were 99.0% (206/208) and 92.8% (193/208), respectively (Fig. 2). There were 13 cases of clinical failure, and seven (3.4%) ultimately required ES owing to adverse events (perforation in four [1.9%] and sepsis in one [0.5%]) and preprocedural obstructive colitis worsened (two patients, 1.0%), whereas scheduled elective surgery was performed in 201 patients (96.6%).

Laparoscopic and open surgeries were performed in 129 (62.0%) and 70 (33.7%) patients, respectively (Table 2). Colectomy with primary anastomosis was performed in 193 patients (92.8%), including diverting stoma in five patients (2.4%). Hartmann’s procedure was performed in 15 patients (7.2%), and the overall stoma creation rate was 9.6% (20/208). The most common complications included bowel obstruction (*n* = 11, including two patients requiring reoperation) and wound infection (*n* = 11), followed by anastomotic leakage (*n* = 4) and enterocolitis (*n* = 3). Nineteen patients were hospitalized for more than 30 days; however, no in‐hospital or 30‐day mortality occurred.

**Table 2:** Surgical procedures and postoperative complications (*n* = 208)

Decompression period, days, median (range, IQR)	17 (0–70, 12–22)
Surgical approach, *n* (%)	
Laparoscopy	129 (62.0)
Open	70 (33.7)
Conversion from laparoscopy to open	9 (4.3)
Surgical procedures, *n* (%)	
Colectomy with primary anastomosis	193 (92.8)
Diverting stoma	5 (2.4)
Hartmann’s procedure	15 (7.2)
Overall stoma creation, *n* (%)	20 (9.6)
Temporary	7 (3.4)
Permanent	13 (6.3)
Postoperative complications,^†^ *n* (%)	All grades
Overall	35 (16.8)
Bowel obstruction	11 (5.3); reoperation 2 (1.0)
Wound infection	11 (5.3)
Anastomotic leakage	4 (1.9)
Enterocolitis	3 (1.4)
Intra‐abdominal abscess	1 (0.5)
Pancreatic fistula	1 (0.5)
Drain infection	1 (0.5)
Mesenteric panniculitis	1 (0.5)
Pulmonary complications	1 (0.5)
Sepsis	1 (0.5)
Renal failure	1 (0.5)
Long hospital stay (over 30 days)	19 (9.1)
30‐day mortality, *n* (%)	0 (0)
Hospital mortality, *n* (%)	0 (0)

^†^ Cases may have overlapping complications.

### Long‐term outcomes

**Figure 3:**
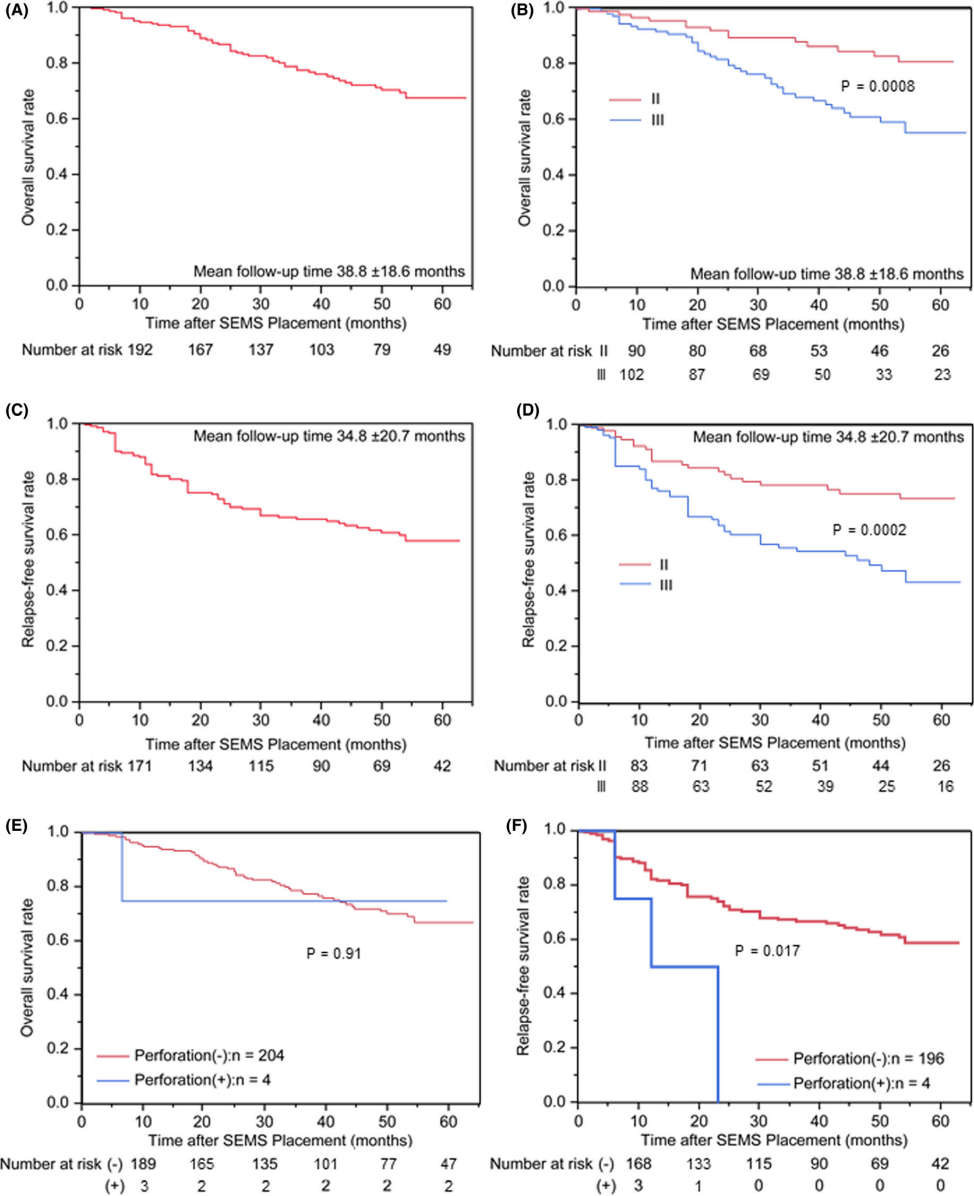
Kaplan–Meier curves for overall survival (OS) and relapse‐free survival (RFS). (A,B) OS of the entire cohort (A) and subgroups of patients with different tumor stages (B) after self‐expandable metallic stent (SEMS) placement (*n* = 208). (C,D) RFS of the entire cohort (C) and subgroups of patients with different tumor stages (D) after SEMS placement (*n* = 200). (E,F) Kaplan–Meier curves for the OS and RFS of patients with apatients were Japanese, with a median agend without perforation. (E) OS of four patients with perforation and 204 patients without perforation. (F) RFS of four patients with perforation and 196 patients without perforation.

The 1‐, 3‐, and 5‐year OS rates were 94.1%, 77.4%, and 67.4%, respectively, during the mean follow‐up period of 38.8 ± 18.6 months (Fig. 3A). The OS rates of patients with stage II and III tumors were 95.8% and 92.8% at 1 year, 88.2% and 68.3% at 3 years, and 81.2% and 55.6% at 5 years, respectively (*P =* 0.0008; Fig. 3B).

After excluding eight patients with insufficient data on recurrence, the 1‐, 3‐, and 5‐year RFS rates of the remaining 200 patients were 81.6%, 65.6%, and 57.9%, respectively, during the mean follow‐up period of 34.8 ± 20.7 months (Fig. 3C). The RFS rates of patients with stage II and III tumors were 86.8% and 77.2% at 1 year, 78.3% and 54.6% at 3 years, and 73.3% and 43.4% at 5 years, respectively (*P =* 0.0002; Fig. 3D).

The overall recurrence rate was 31.0% (62/200), and the primary recurrence sites were locoregional and distant metastases in 30 (15%) and 39 (19.5%) patients, respectively (Table 3). The most common locoregional recurrence occurred in the peritoneum (26 patients, 13.0%), whereas other recurrences occurred in regional lymph nodes, local sites (three patients each, 1.5%), and anastomosis (one patient, 0.5%). Conversely, the most common distant metastases occurred in the liver (27 patients, 13.5%), followed by the lungs (15 patients, 7.5%).

**Table 3:** Primary recurrence sites after surgery (*n* = 200)

Site	Number of recurrences, *n* (%)
Overall^†^	62 (31.0)
Locoregional recurrence	30 (15.0)
Regional lymph node	3 (1.5)
Local	3 (1.5)
Anastomosis	1 (0.5)
Peritoneum	26 (13.0)
Distant metastases	39 (19.5)
Lymph node (not regional)	4 (2.0)
Liver	27 (13.5)
Lung	15 (7.5)
Bone	3 (1.5)
Brain	1 (0.5)

^†^ Including overlapping cases.

### Subgroup analysis for long‐term outcomes in patients with or without perforation

Among the 208 patients, perforation occurred in four patients (1.9%). Table 4 summarizes the details of these four patients, all of whom were treated with ES. The subgroup analysis to compare long‐term outcomes in patients with and without perforation revealed that the 1‐, 3‐, and 5‐year OS rates were 94.5%, 77.5%, and 67.3%, respectively, in 204 patients without perforation and 75.0%, 75.0%, and 75.0%, respectively, in four patients with perforation, with no significant intergroup differences (*P =* 0.91; Fig. 3E). The 1‐, 3‐, and 5‐year RFS rates were 82.3%, 66.6%, and 58.7%, respectively, in 196 patients without perforation, whereas the 1‐year RFS rate was 50.0% in the four patients with perforation. The RFS was significantly poorer in patients with perforation than in those without perforation (*P =* 0.017; Fig. 3F). Moreover, in the multivariate Cox regression analysis, perforation (hazard ratio [HR] 3.31; 95% confidence interval [CI] 1.03–10.71, *P =* 0.045) as well as TNM staging (HR 2.52 for III vs. II; 95% CI 1.23–4.18, *P =* 0.0003) were significantly associated with RFS and identified as independent factors affecting RFS (Table 5).

**Table 4:** Characteristics of patients with perforation (*n* = 4)

Case no.	Cause of perforation	Age (years)	Sex	Perforation time after SEMS placement (days)	Tumor location	Perforation site	Treatment	Primary recurrence site
1	Guidewire	60	M	0	S	S	Emergency surgery (Hartmann’s procedure)	Liver
2	Proximal bowel (obstructive colitis)	86	M	2	A	C	Emergency surgery (Hartmann’s procedure)	Peritoneum
3	Stent	81	F	2	R	R	Emergency surgery (Hartmann’s procedure)	Lung
4	Stent	71	M	19	S	S	Emergency surgery (Hartmann’s procedure)	No recurrence

A, ascending colon; C, cecum; F, female; M, male; R, rectum; S, sigmoid colon.

**Table 5:** Univariate and multivariate Cox regression analyses for relapse‐free survival (*n* = 200)

Variable	Univariate		Multivariate*	
	HR (95% CI)	*P*	HR (95% CI)	*P*
Age (≥72 vs. <72)	1.04 (0.65–1.64)	0.88		
Sex (male vs. female)	1.27 (0.80–2.02)	0.32		
PS (2–4 vs. 0 or 1)	1.45 (0.78–2.69)	0.24		
CROSS (0 vs. 1–4)	1.28 (0.80–2.05)	0.30		
Tumor location (right colon vs. left colon or rectum)	1.19 (0.70–2.00)	0.52		
TNM staging (III vs. II)	2.50 (1.51–4.13)	0.003	2.52 (1.23–4.18)	0.0003
Surgical approach (laparoscopy vs. open)	1.01 (0.63–1.62)	0.98		
Postoperative complication	1.39 (0.79–2.45)	0.26	1.53 (0.86–2.71)	0.14
Perforation	3.76 (1.17–12.10)	0.027	3.31 (1.03–10.71)	0.045

* Model *P*‐value <0.001.

CI, confidence interval; CROSS, ColoRectal Obstruction Scoring System; HR, hazard ratio; PS, performance status; TNM, tumor–node–metastasis.

## DISCUSSION

In this study, patients treated with BTS colonic stenting for stage II and III obstructive colon cancer had reasonably high OS and RFS rates. The short‐term outcomes were excellent with BTS colonic stenting using a standardized SEMS insertion method, partially attributable to the high technical success rate and low perforation rate. However, despite the extremely low perforation rate, RFS was significantly poorer in patients with perforation than in those without perforation, and perforation was also identified as an independent factor affecting RFS.

The OS rates in this study (1‐year: 94.1%, 3‐year: 77.4%, 5‐year: 67.4%) were not only higher than those previously reported for BTS colonic stenting but also comparable to those reported in patients undergoing ES (Table 6). The 3‐ and 5‐year OS rates in this study were higher than the previously reported rates of BTS colonic stenting (51–76%^8^, ^17^, ^18^, ^19^, ^20^, ^21^ and 30–57.1%,^19^, ^20^, ^21^ respectively). The 3‐ and 5‐year OS rates of BTS colonic stenting in this study were nearly the same or higher than those of ES reported in previous studies (45–78%^8^, ^17^, ^18^, ^19^, ^20^, ^21^ and 35–67%,^19^, ^20^, ^21^ respectively). One possible explanation for these satisfactory long‐term outcomes is the use of a standardized SEMS insertion method. As part of an extensive program implemented by JCSSPRG, periodic meetings were hosted and a website was created to spread awareness and information regarding the procedural safety of SEMS placement. This approach provided participating endoscopists with access to the technical knowledge on the standardized procedure before the study initiation. These standardization practices have likely contributed to the excellent short‐term outcomes of SEMS placement, especially the high technical success rate (99.0%) and low perforation rate (1.9%). Considering that a low technical success rate (<90%) and high perforation rate (>8%) could lead to poor long‐term prognoses,^22^ BTS colonic stenting using a standardized SEMS insertion method, which resulted in excellent short‐term outcomes, may be effective for achieving satisfactory long‐term outcomes.

**Table 6:** Overall and relapse‐free survival rates in the current and previous studies

	Study design	Setting	*n*	3‐year OS	5‐year OS	3‐year RFS
Current study	Prospective cohort study	BTS	208	77.4%	67.4%	65.6%
Amelung *et al*. 2019^†^ ^,^ ^17^	Retrospective ITT analysis	ES	444	68.3%		
		BTS	222	74.0%		
Arezzo *et al*. 2017^18^	RCT	ES	59	69%^‡^		62%^‡^
		BTS	56	64%^‡^		58%^‡^
Ho *et al*. 2017^19^	Retrospective ITT analysis	ES	40	45%^‡^	35%^‡^	
		BTS	62	60%^‡^	54%^‡^	
Sloothaak *et al*. 2014^8^	Follow‐up data of RCT	ES	32	78%^‡^		
		BTS	26	62%^‡^		
Sabbagh *et al*. 2013^20^	Retrospective ITT analysis	ES	39	74%	67%	
		BTS	48	51%	30%	
Tung *et al*. 2013^21^	Follow‐up data of RCT	ES	24	78%^‡^	42.8%	
		BTS	24	76%^‡^	57.1%	

^†^ In this study 10% of patients in the cohort presented with distant metastases.

^‡^ Approximate data from figures.

BTS, bridge‐to‐surgery; ES, emergency surgery; ITT, intention‐to‐treat; OS, overall survival; RCT, randomized controlled trial; RFS, relapse‐free survival.

The RFS (1‐year: 81.6%, 3‐year: 65.6%, 5‐year: 57.9%) and overall recurrence (31.0%) rates in the present study were also comparable to those of recent studies (Table 6). In the ESCO randomized trial, Arezzo *et al*.^18^ reported 3‐year RFS rates of approximately 58% and 62% and overall recurrence rates of 30.3% and 33.9% in the BTS stenting and ES groups, respectively. In a propensity‐matched analysis, Amelung *et al*.^17^ reported overall recurrence rates of 31.5% and 38.5% in the BTS stenting and ES groups, respectively. These RFS and recurrence rates, especially in the ES groups, were similar to those reported in our study. However, according to the updated European Society of Gastrointestinal Endoscopy guidelines,^9^ although there is no difference in OS between BTS stenting and ES, concerns remain regarding higher overall recurrence with BTS stenting. Interestingly, most of the data presented in several relevant meta‐analyses, which indicated poor recurrence rates associated with BTS stenting, were obtained from older studies with poor overall short‐term outcomes and high perforation rates.^5^, ^6^, ^23^ However, more recent studies have reported no difference in overall recurrence rates between BTS colonic stenting and ES.^17^, ^18^ Compared with data from previous studies involving ES, our data revealed similar RFS and overall recurrence rates. As mentioned above with regard to OS, we believe that the standardized SEMS placement might contribute to the comparably high RFS and low overall recurrence rates in this study by providing excellent short‐term outcomes, especially low perforation rates. In fact, in the subgroup analysis, despite the very low perforation rate, RFS was significantly poorer in patients with perforation than in those without perforation; moreover, perforation was also identified as an independent factor affecting RFS. Therefore, reducing the perforation rate under standardized conditions may be an effective approach to improve long‐term outcomes.

One of the most common primary recurrence sites in our study was the peritoneum, observed in 26 patients (13.0%), with a much higher rate than that observed in patients with stage II and III colorectal cancer in the Japanese Society for Cancer of the Colon and Rectum registry (3–4%).^24^ This observation might nevertheless be a feature of obstructive colorectal cancer, since higher peritoneal recurrence was reported not only in patients undergoing BTS stenting but also those undergoing ES.^18^, ^25^ Conversely, the oncological impact of SEMS expansion as well as perforation owing to SEMS placement is concerned with the possibility of attribution to peritoneal recurrence.^26^ SEMS expansion may contribute to dissemination of cancer cells into the systemic circulation.^27^, ^28^ Additionally, the perineural invasion rate of a primary tumor, which can result in deeper metastatic invasion, was higher after BTS colonic stenting than after ES.^29^ In contrast, Matsuda *et al*.^30^ reported that mechanical compression by SEMS might suppress cancer cell proliferation. Therefore, the impact of SEMS expansion on cancer cell dissemination remains controversial and requires further investigation because SEMS placement inevitably results in mechanical compression despite standardized procedures.

This study has several limitations. First, this was a nonrandomized study with no control group, limiting inferences that could be made regarding causality. Second, as this was an observational study, the details of the treatment strategy (e.g. duration between SEMS placement and elective surgery, implementation of adjuvant chemotherapy) were determined by each facility, which might have led to bias in the clinical outcomes. Third, the study was conducted solely in Japan using only one type of SEMS device (WallFlex), limiting its generalizability. In addition, Japanese endoscopists now prefer to use lower axial force SEMS devices rather than WallFlex. Nevertheless, this was one of the largest multicenter prospective studies in the field, including 46 hospitals and 208 participants, and WallFlex is the most commonly used SEMS device globally. Therefore, the satisfactory long‐term outcomes in the present study underscore the importance of standardized SEMS insertion as a BTS approach.

In conclusion, this large, prospective, multicenter study of Japanese patients with stage II and III obstructive colon cancer revealed satisfactory long‐term outcomes of BTS colonic stenting by using a standardized SEMS insertion method. This emphasizes the importance of standardized BTS SEMS placement in short‐term as well as long‐term outcomes, which might be specifically owing to reduced perforation rates.

## CONFLICT OF INTEREST

T.K. received personal fees from Boston Scientific Japan and Century Medical, Inc. T.M. received a personal fee from Boston Scientific Japan. H.I. received donations and fees from Boston Scientific Japan, Century Medical, Inc., and Taewong Medical. S.S. received personal fees from Boston Scientific Japan, Century Medical, Inc., Cook Japan, Japan Lifeline Co., Ltd., and Kawasumi Laboratories, Inc. K.K. received personal fees from Century Medical, Inc. and Olympus Medical Systems. Y.S. received grants and personal fees from Boston Scientific Japan, Century Medical, Inc., and Olympus Medical Systems. The other authors declare no conflict of interest for this article.

## FUNDING INFORMATION

This study was conducted with Japan Gastroenterological Endoscopy Society funding support and the Japan Colonic Stent Safe Procedure Research Group membership dues.

## Supporting information


**Appendix S1** Safe procedure concepts for colonic self‐expandable metallic stent (SEMS) placement recommended by the Japan Colonic Stent Safe Procedure Research Group.
