# Supplementary material for: Long‐term outcomes of standardized colonic stenting using WallFlex as a bridge to surgery: Multicenter prospective cohort study
Source: Dig Endosc. 2021 Oct 1;34(4):840–9. doi: 10.1111/den.14137 (PMC9293325; doi:10.1111/den.14137)
Supplement: Supplementary file 1 — Appendix S1 Safe procedure concepts for colonic self‐expandable metallic stent (SEMS) placement recommended by the Japan Colonic Stent Safe Procedure Research Group. [file DEN-34-840-s001.docx]

**Appendix S1**

Safe procedure concepts for colonic self-expandable metallic stent (SEMS) placement recommended by the Japan Colonic Stent Safe Procedure Research Group (for further details, refer to <https://colon-stent.com/001_mainpage_en.html>).

1. SEMS placement should be attempted as soon as possible after the onset of intestinal obstruction. Prophylactic SEMS placement should never be performed.
2. The procedure is performed in a fluoroscopy room.
3. Colonoscopy is performed under carbon dioxide insufflation using a colonoscope with a large-bore (3.7 mm) channel (forceps channel) (CF-Q240I, CF-H260AI, or CF-HQ290L/I; Olympus Corporation, Tokyo, Japan) for the insertion of a WallFlex enteral colonic stent (Boston Scientific, Marlborough, MA, USA).
4. Distal end of the stenotic lesion should be marked with a metal clip.
5. Additional procedures, such as biopsy, should be kept to an absolute minimum to secure the best possible visual field.
6. SEMS is deployed after a guidewire has been sufficiently advanced into the proximal end of the stenosis.
7. The guidewire used to pass through the stenosis must be narrow (0.025 in [0.635 mm] or 0.035 in [0.9 mm]), and a sheath (an endoscopic retrograde cholangiopancreatography [ERCP] cannula or a SwingTip [Olympus Medical Systems, Tokyo, Japan] in cases of difficult insertion) must be used, similar to that performed during ERCP.
8. In cases of difficulty in advancing the guidewire through the stenosis, an attachment is connected to the distal end of the scope, or the colonoscope is exchanged for a small-diameter endoscope or upper gastrointestinal endoscope, and the procedure is attempted again.
9. If the guidewire can be advanced through the stenosis, the sheath is advanced into the proximal end of the stenosis and imaging is performed to confirm the placement of the guidewire within the intestinal lumen and to measure the length of the stenosis.
10. Balloon or bougie dilatation of the stenotic region should not be performed.
11. SEMS placement can be performed after the guidewire has been sufficiently advanced into the proximal end of the stenosis and is parallel to the intestinal wall without flexion or rolling.
12. If the visual field is disrupted by hemorrhage after guidewire insertion, blood should be removed by rinsing with water or the guidewire may be temporarily withdrawn. Advancement must not be attempted under any circumstance when the visual field is inadequate.
13. After its insertion into the proximal end of the stenosis, SEMS gradually expands and assumes the proper position while traction is applied to the delivery system and the endoscope.
14. Markers are used to confirm SEMS position, and SEMS is expanded by the inherent release method.
15. After the procedure is completed, fluoroscopic or plain abdominal radiography should be performed to confirm the absence of free air, which indicates intestinal injury.
16. Patients should be followed by abdominal radiography and physical examination for two days after SEMS placement.
